# *Caralluma tuberculata* N.E.Br Manifests Extraction Medium Reliant Disparity in Phytochemical and Pharmacological Analysis

**DOI:** 10.3390/molecules26247530

**Published:** 2021-12-13

**Authors:** Muhammad Waleed Baig, Madiha Ahmed, Nosheen Akhtar, Mohammad K. Okla, Bakht Nasir, Ihsan-Ul Haq, Jihan Al-Ghamdi, Wahidah H. Al-Qahtani, Hamada AbdElgawad

**Affiliations:** 1 Department of Pharmacy, Faculty of Biological Sciences, Quaid-i-Azam University, Islamabad 45320, Pakistan; mwbg7@yahoo.com (M.W.B.); pharmacist_madiha@hotmail.com (M.A.); bakhtnasir61@yahoo.com (B.N.); 2 Shifa College of Pharmaceutical Sciences, Shifa Tameer-e-Millat University, Islamabad 45320, Pakistan; 3 Department of Biological Sciences, National University of Medical Sciences, Rawalpindi 46000, Pakistan; nosheenakhtar@numspak.edu.pk; 4 Botany and Microbiology Department, College of Science, King Saud University, P.O. Box 2455, Riyadh 11451, Saudi Arabia; Okla103@yahoo.com (M.K.O.); jalghamdi@ksu.edu.sa (J.A.-G.); 5 Department of Food and Nutrition, College of Food and Agriculture Sciences, King Saud University (KSU), Riyadh 11451, Saudi Arabia; wahida@ksu.edu.sa; 6 Integrated Molecular Plant Physiology Research, Department of Biology, University of Antwerp, 2020 Antwerpen, Belgium; hamada.abdelgawad@uantwerpen.be

**Keywords:** *Caralluma*, phytochemical, antimicrobial, cytotoxicity, antioxidant, protein kinase

## Abstract

Solubility of phytoconstituents depends on the polarity of the extraction medium used, which might result in the different pharmacological responses of extracts. In line with this, ethnomedicinally important food plant (i.e., *Caralluma tuberculata* extracts) have been made in fourteen distinct solvent systems that were then analyzed phytochemically via total phenolic amount estimation, total flavonoid amount estimation, and HPLC detection and quantification of the selected polyphenols. Test extracts were then subjected to a battery of in vitro assays i.e., antioxidants (DDPH scavenging, antioxidant capacity, and reducing power estimation), antimicrobial (antibacterial, antifungal, and antileishmanial), cytotoxic (brine shrimps, THP-1 human leukemia cell lines and normal lymphocytes), and protein kinase inhibition assays. Maximum phenolic and flavonoid contents were computed in distilled water–acetone and acetone extracts (i.e., 16 ± 1 μg/mg extract and 8 ± 0.4/mg extract, respectively). HPLC-DAD quantified rutin (0.58 µg/mg extract) and gallic acid (0.4 µg/mg extract) in methanol–ethyl acetate and methanol extracts, respectively. Water–acetone extract exhibited the highest DPPH scavenging of 36 ± 1%. Total reducing potential of 76.0 ± 1 μg/mg extract was shown by ethanol chloroform while maximum total antioxidant capacity was depicted by the acetone extract (92.21 ± 0.70 μg/mg extract). Maximal antifungal effect against *Mucor sp.*, antileishmanial, brine shrimp cytotoxicity, THP-1 cell line cytotoxicity, and protein kinase inhibitory activities were shown by ethyl acetate-methanol (MIC: 50 µg/disc), n-hexane (IC_50_: 120.8 ± 3.7 µg/mL), ethyl acetate (LD_50_: 29.94 ± 1.6 µg/mL), distilled water–acetone (IC_50_: 118 ± 3.4 µg/mL) and methanol–chloroform (ZOI: 19 ± 1 mm) extracts, respectively. Our findings show the dependency of phytochemicals and bioactivities on the polarity of the extraction solvent and our preliminary screening suggests the *C. tuberculata* extract formulations to be tested and used in different ailments, however, detailed studies remain necessary for corroboration with our results.

## 1. Introduction

The “one disease one drug” concept in the modern drug discovery system is at the verge of losing its importance because of the development of complex, interrelated, and multitargeted disease outcomes. In this scenario, plant extracts have emerged as a source to target multiple diseases at once. A plethora of secondary metabolites in extracts work synergistically, and rarely is a unit phytochemical entity responsible for their pharmacological response [1]. Broad interdisciplinary approaches play a pivotal role in determining the full medicinal potential of any plant. These approaches include phytochemistry, bioassays, and mechanism identification strategies, in addition to ethnopharmacology [2].

Plants including vegetables can be exercised as therapeutic agents. Remedial properties of vegetables and fruits have been protected in the form of ethno-botanical tradition and as ancient heritage [3]. According to the WHO, people are eating less than 20–50% of vegetables, which has raised many concerns [4]. One third of the population have lifelong and serious health issues such as diabetes, allergy, fatigue, arthritis, heart problems, and cancer. A long list of phytonutrients in vegetables is beneficial in disorders such as anthocyanidins for myocardial infarction, catechins for chemoprevention, glucosinolates to boost immunity, phenols for inflammation, thiols for infections, and tochopherols to lowering cholesterol [3]. From head pain to cardiovascular diseases, plant based (vegetables and fruits) consumption is considered to be healthy in curing or preventing ailments [5] and scientific evidence supports this claim.

Genus *Caralluma* R. Br. of the Apocynaceae family includes 120 species, all of which are xerophytes and branched herbs [6]. *Caralluma tuberculata* N. E. Br. is an edible, juicy, leafless stiff plant that is propagated in dry, undomesticated regions of Pakistan and its neighboring countries Saudi Arabia, Nigeria, and Iran [7]. Ethnomedicinal applications include its usage in dysentery, jaundice, constipation, stomach pain, freckles and pimples, hepatitis B and C, diabetes, blood purification, liver ailments, rheumatism, febrifuge, hypertension, gastric problems, paralysis, inflammation, and cancer [8]. A literature survey showed antioxidant, antibacterial [7], hypolipidemic [9], anti-hyperglycemic [10], and in vitro anticancer potentials [11].

A comprehensive and retrospective review of the literature showed that there is still a gap for further research. No systematized studies have been performed, which encompass a chain of bioassays to evaluate the maximum therapeutic potential of *C. tuberculata*. Solvents that are routinely used for extraction (i.e., methanol and ethanol) do not extract all bioactive constituents; therefore, therapeutic profiling is not complete using a unipolarity extraction system approach. The current study is an attempt to provide a systemized and comparative report of fourteen wide polarity range extracts of *C. tuberculata* in varied solvents, either alone or in 1:1 combination, on a battery of antioxidant and biological assays (i.e., phosphomolybdenum based antioxidant capacity, reducing potential, HPLC fingerprinting, antifungal, antileishmanial, THP-1 cytotoxicity, and protein kinase inhibition assays).

## 2. Material and Methods

The methodology followed in the study are standard protocols that have been cited properly.

### 2.1. Reagents and Solvents

Reagent and solvent utilized were of analytical grade and bought from Sigma-Aldrich, USA and Germany. Solvents employed in the extraction process were acetone, methanol, ethyl acetate, ethanol, n-hexane, and chloroform while dimethylsulfoxide (DMSO) was used for sample preparation. Cefixime, clotrimazole ciprofloxacin, amphotericin B, and vincristine were standard drugs used. Remaining chemicals and reagents used in the research were: FC = Folin–Ciocalteu reagent; AlCl_3_ = aluminum chloride; FeCl_3_ = ferric chloride; TCA = trichloroacetic acid; DPPH = 2,2-diphenyl-1-picryl-hydrazyl-hydrate; sea salt; PBS = phosphate buffer saline; NaH_2_PO_4_ = monosodium dihydrogen phosphate; kaempferol; catechin; myricitin; gallic acid; quercetin; and rutin were purchased from Merck (Darmstadt, Germany) unless otherwise stated.

### 2.2. Plant Collection and Identification

The plant material (aerial portion) was collected from Kalabagh region, district Miyanwali, Punjab, Pakistan in June 2014. Prof. Dr. Rizwana Aleem Qureshi recognized and verified the field gathered plant. Dried plant was reserved in the Herbarium of Medicinal plants, Quaid-i-Azam University Islamabad under herbarium number 498.

### 2.3. Extraction

The putrefied plant part was removed, washed with tap water, dried in a shaded area, and finally pulverized. An amount of 50 g powder was weighed in each Erlenmeyer flask and macerated in the respective solvents with episodic shaking and ultrasonication. After three days, marc was separated and filtered (Whatman No. 1 filter paper). Finally, the extract was concentrated using a rotary evaporator (Buchi, 150 Switzerland) and dried to obtain fourteen different crude extracts.

Extract recovery was calculated as:

(1) Extract recovery=weight of dried extract/weight of powdered plant used ×100,

### 2.4. Phytochemical Analysis

#### 2.4.1. Total Phenolics Content Estimation (TPC)

The Folin–Ciocalteu method was adopted. DMSO and gallic acid were used as positive and negative controls. The resultant phenolics (TPC) after triplicate analysis were expressed as µg gallic acid equivalent (GAE) per mg extract [12].

#### 2.4.2. Total Flavonoids Content Estimation (TFC)

DMSO and quercetin (0, 2.5, 5, 10, 20 µg/mL) were used as negative and positive controls, respectively. After triplicate analysis, a calibration curve was drawn and the final TFC content was expressed as µg QE (quercetin equivalent) per mg extract of plant [13].

#### 2.4.3. HPLC-DAD Quantitative Analysis

HPLC-DAD analysis was performed with slight adaptations [14]. The HPLC (Agilent Chem Station 1200 series, USA) system had a diode array DAD detector (Agilent technologies, Germany) along with a C8 analytical column. Standard solutions (50 µg/mL methanol) and sample solutions (10 mg/mL methanol) were freshly made and filtered using a 0.2 µm sartolon polyamide membrane filter. Available reference standards included kaempferol, caffeic acid, quercetin, rutin, myricitin, gallic acid, catechin, and apigenin. Two mobile phases, A and B, were used where mobile phase A had methanol:water:acetic acid:acetonitrile (10:85:1:5) and mobile phase B had acetic acid:acetonitrile:methanol (1:40:60). The flow rate was 1 mL/min. Twenty µL of the sample was injected into the column. Every sample analysis was followed by a 5 min column reconditioning phase. The gradient volume of mobile phase B was adjusted as shown below (Table 1).

Absorbance was measured at four different wavelengths for the respective compounds (i.e., 279 nm for catechin, 257 nm for gallic acid and rutin, 368 nm for quercetin kaempferol, and myricitin, and 325 nm for caffeic acid).

### 2.5. Biological Evaluation

#### 2.5.1. Antioxidant Assays

Free radical scavenging assay (DPPH assay)

An established protocol was followed. Sample (4 mg/mL), ascorbic acid, and DMSO were used as positive and negative controls, respectively [15].

#### 2.5.2. Total Antioxidant Capacity Determination

A well-established protocol was followed. Test samples, positive (ascorbic acid) and negative (DMSO) controls were used. Results are expressed as the number of µg equivalents of ascorbic acid per mg of dry extract (i.e., µg AAE/ mg extract) [15].

#### 2.5.3. Total Reducing Power Estimation

A previously reported methodology was followed. DMSO and ascorbic acid as the negative and positive controls, respectively, were used. Reducing power was expressed as µg ascorbic acid equivalent (AAE) per mg extract after triplicate analysis [12].

#### 2.5.4. Antimicrobial Assays

Test extracts were tested against five bacterial strains (i.e., *B. subtilis* (ATCC-6633), *S. aureus* (ATCC-6538), *K. pneumoniae* (ATCC-1705), *E. coli* (ATCC-25922), and *P. aeruginosa* (ATCC-15442) while fungal strains include *F. solani* (FCBP-0291), *Mucor sp.* (FCBP-0300), *A. flavus* (FCBP-0064), *A. fumigatus* (FCBP 66), and *A. niger* (FCBP-0198). The agar disc diffusion method was adopted. To prepare lawn on agar plates, 50 µL volume of refreshed culture was used. Positive and negative controls included cefixime, ciprofloxacin, clotrimazole, and DMSO. To a sterile filter paper disc, 5 µL (200 µg/disc) of test extract was applied, which was then settled on seeded agar plates and were incubated for 24 h. After the given time, emergence of the zone of inhibition around the disc was checked to the nearest mm with vernier calipers. Test extracts that gave ≥12 mm meaningful inhibition zones were further evaluated for MICs using the same method as in the antifungal assay [15]. Bacterial inoculum was made in pre-autoclaved nutrient broth under sterile conditions, density being adjusted to 5 × 10^2^ CFU/mL, approximately. Final concentrations of test samples (200 66.66, 22.22, and 7.4 µg/mL) in corresponding wells were made. A total of 190 µL bacterial culture was added to each well, incubated for 30 min at 37 °C, zero time reading taken at 600 nm, again incubated for 24 h at 37 °C, absorbance checked, and results calculated as:

(2) $$\%\;\mathrm{Growth}\;\mathrm{inhibition}=1-\;T_{s}\;/\;T_{c}\times 100,$$

T_s_ and T_c_ = turbidity of the sample and negative control, respectively.

#### 2.5.5. In Vitro Antileishmanial Potential Determination

Serial dilutions of test samples (40 mg/mL DMSO) were prepared in 96-well plates so that final concentrations were 200, 66.66, and 22.22 µg/mL, respectively. To each well, 1 × 10^6^ *L. tropica* kwh23 promastigotes were added and incubated at 25 °C for 72 h. Surviving promastigotes were counted using a light microscope. The same procedure was performed on Amphoterecin-B (positive control) and 1% DMSO in PBS (negative control). Finally, IC_50_ was calculated [15].

#### 2.5.6. Cytotoxicity Assays

##### Lethality Testing in Brine Shrimps

Freshly hatched *Artemia salina* larvae in sea water were picked and transferred to each well of a 96-well plate. Calculated volume of four concentrations (200, 100, 50, and 25 µg/mL) of test samples was added to the corresponding wells. Positive and negative control wells include doxorubicin (4 mg/mL) and DMSO (<1%). Volume was made up to 300 µL by adding more sea water. Dead larvae that settled at the base of the wells were counted with a microscope after 24 h. This procedure was repeated twice. Finally, the LC_50_ of test samples was calculated [15].

#### 2.5.7. Cytotoxicity Assay Using THP-1 Human Leukemia Cell Line

Depending upon the system suitability, a slightly different approach was followed as described by [15]. The THP-1 cell line was purchased from ATCC 10801 University Boulevard Manassas, VA 20110-2209 USA. Culturing involved THP-1 (ATCC-TIB202) human leukemia cell lines in RPMI 1640 buffered medium (pH 7.4) supplemented with fetal bovine serum (10%). Incubation was conducted with conditions maintained at: 5% CO_2_ and 37 °C. Seeding density was adjusted to 5 × 10^5^ cells/mL. A total of 190 µL of culture was added thereafter, and 10 µL (test samples (1% DMSO) in PBS) was added to the respective wells. The plate was then allowed to incubate (5% CO_2_) for 72 h at 37 °C. The assay was also performed on 4 mg/mL DMSO solutions of 5-florouracil and vincristine and 1% DMSO in PBS (negative control). Surviving cells were counted by putting culture on an improved Neubauer chamber and observed under microscope, after which IC_50_ was calculated.

#### 2.5.8. Cytotoxicity Assay Using Isolated Human Lymphocytes

Blood (3 mL) was collected via venipuncture from a healthy donor and was diluted with equal proportion of PBS. It was carefully layered over histopaque (2 mL) and centrifuged for 20 min at 800 g. A buffy coat layer was separated, added into 5 mL PBS, and centrifuged (4 min; 350 rpm). The formed pellet was suspended in RPMI-1640. For analysis, 20 µL (sample, positive, and negative control) was exposed to 180 µL of lymphocyte (1 × 10^5^ cells/mL) suspension. Incubation was carried out for one day in an incubator at 37 °C. Phytohaemagglutinin was added to stimulate lymphocyte growth [13]. The study was approved by the Institutional Review board of Quaid-i-Azam University (letter number #BEC-FBS-QAU2019-135A). Written informed consent was obtained from the participant.

#### 2.5.9. Protein Kinase Inhibition Assay

Refreshed *Streptomyces* culture obtained after 24 h incubation in tryptic soy broth was used for the growth in ISP4 media plates. Soaked paper (negative (DMSO), positive (surfactin), and sample (extracts; 100 µg/disc) discs were put on sterile and freshly inoculated plates. Display of bald zones surrounded discs after 72 h incubation time was interpreted as protein kinase inhibitory activity. Zones were noted by vernier calipers up to the nearest mm [15].

#### 2.5.10. Statistical Analysis

Statistical analysis software included Statistx 8.1 (ANOVA; analysis of variance), table cure 2D (IC50 and LD50) v 5.01, Graph Pad Prism (significance level at *p* < 0.05), and Origin 8.5 (graphical representation after triplicate analysis of each experiment).

## 3. Results

The undertaken study was designed in a manner to evaluate phytochemical and biological analysis of *C. tuberculata* using 14 different solvent systems. Extract yield ranged between 25.16% w/w (for distilled water) and 0.86% (for n-hexane) (Table 2).

### 3.1. Phytochemical Analysis

**Figure 1:**
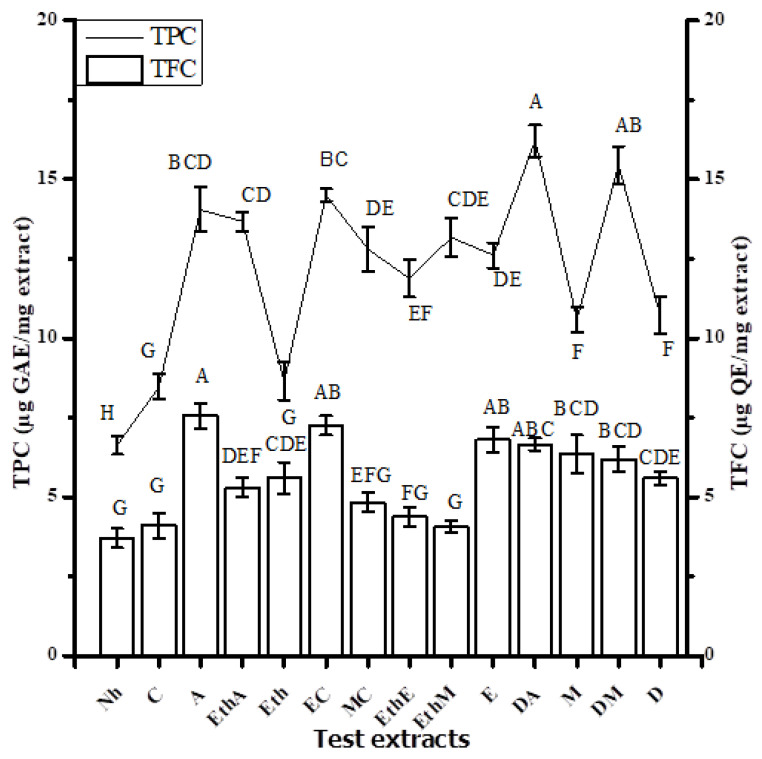
TPC (total phenolic content; mean ± standard deviation) and TFC (total flavonoid contents; mean ± standard deviation) are displayed after triplicate investigation. A–H represent the significance of results. Samples that display different alphabets were significantly different at *p* < 0.05.

**Figure 2:**
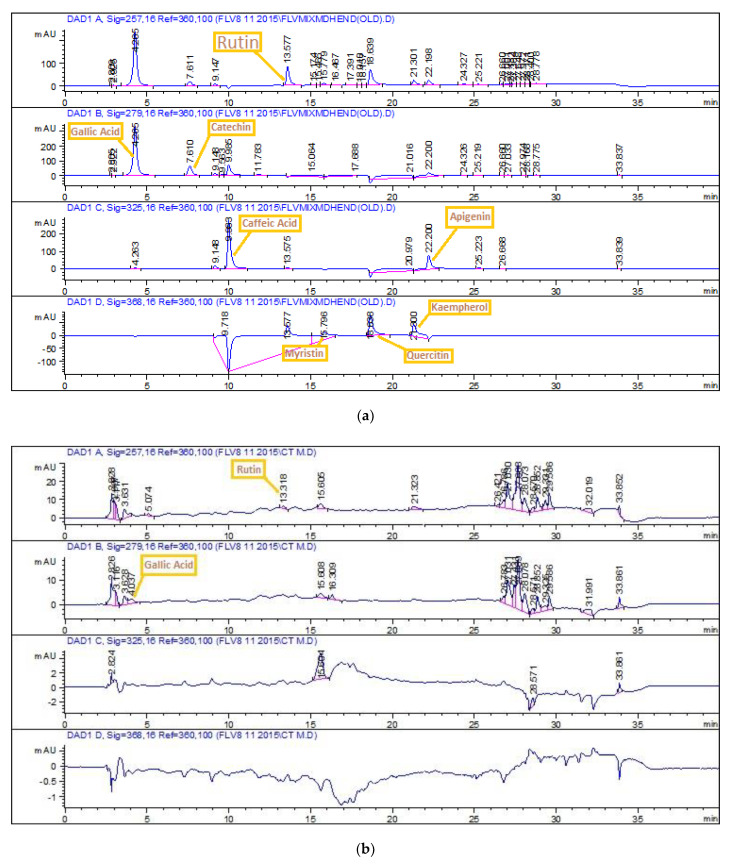

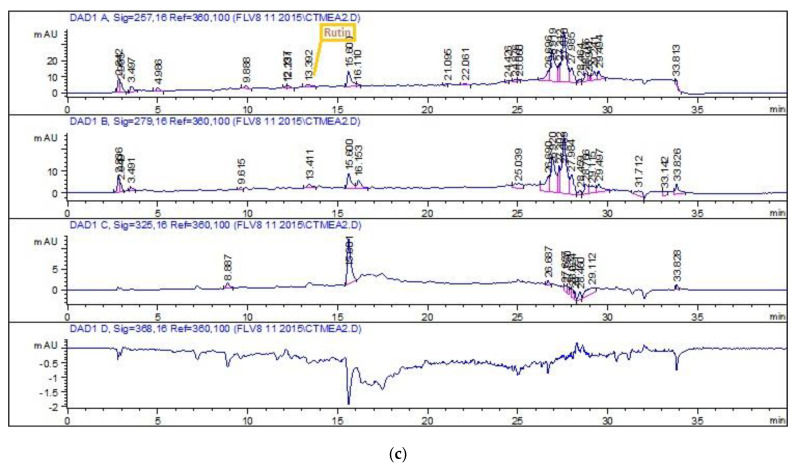
RP HPLC chromatograms of (**a**) standard polyphenols, (**b**) methanol, and (**c**) ethyl acetate–methanol crude extracts of *C. tuberculata*.

Maximum TPC value was quantified in DA (16 ± 1) (i.e., in µg GAE/mg extract) followed by DM and A with corresponding values (i.e., 15.43 ± 0.60 and 14.04 ± 0.72 µg GAE/mg extract, respectively) (Figure 1). The highest TFC value was quantified in A (i.e., 8 ± 0.39 µg QE/mg extract), followed by EC and E test extracts with corresponding values 9 (i.e., 7.25 ± 0.33 and 6.80 ± 0.40 µg QE/mg extract, respectively) (Figure 1). HPLC chromatograms of standards and detected compounds are shown in Figure 2. Calculated results signify the presence of rutin in EthM extract with a value of 0.58 µg/mg extract followed by M extract with a value of 0.51 µg/mg extract (Table 3). A significant amount of gallic acid was found to be present in the M extract with a value of 0.4 µg/mg extract.

### 3.2. Biological Evaluation

#### 3.2.1. Antioxidant Assays

**Figure 3:**
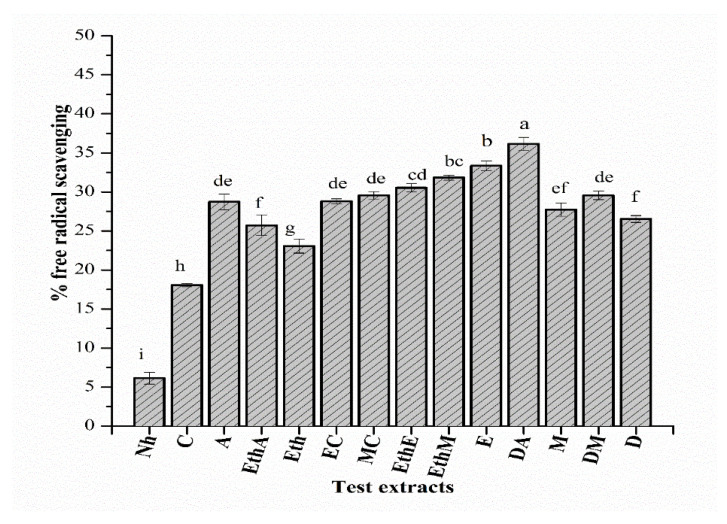
Percent free radical scavenging activity (mean ± standard deviation) after triplicate investigation is shown. a–i represent the significance of results. Samples that display different alphabets were significantly different at *p* < 0.05.

**Figure 4:**
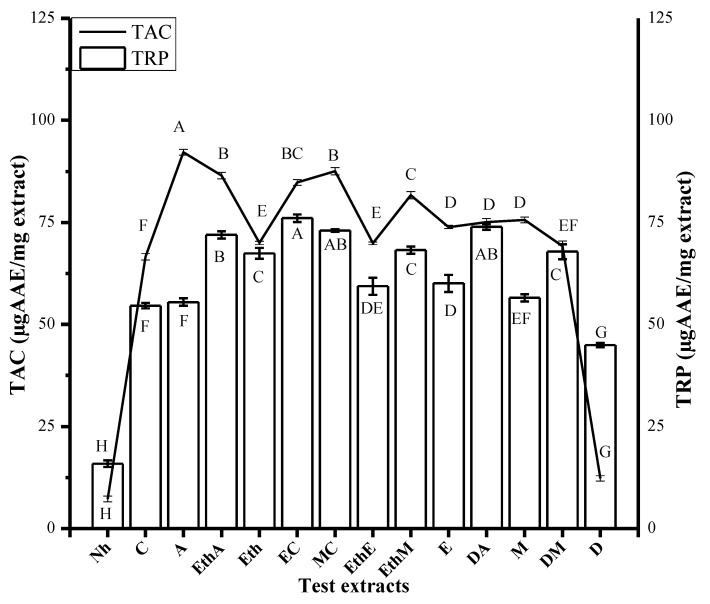
TAC (total antioxidant capacity; mean ± standard deviation) and TRP (total reducing power; mean ± standard deviation) results are displayed after experimenting thrice. A–F represent the significance of results. Samples that display different alphabets were significantly different at *p* < 0.05.

Among the tested extracts, the DA (200 µg/mL) extract exhibited the maximum free radical scavenging potentiality (i.e., 36.13% ± 0.84), followed by A and EthA with corresponding values of 33.712% ± 1 and 33.712% ± 1.32, respectively (Figure 3). Assay findings suggest marked antioxidant capacity in A (92.21 ± 0.70 µg AAE/mg extract), followed by MC and EaA with corresponding values of 87.53 ± 0.9 and 86.5 ± 0.80 µg AAE/mg extract, respectively (Figure 4). Computed results in the total reducing power assay symbolize meaningful activity in EC (76.01 ± 0.90 µg AAE/mg extract), followed by DA (73.94 ± 0.70 µg AAE/mg extract) and EaA (71.92 ± 0.93 µg AAE/mg extract) (Figure 4).

#### 3.2.2. Antimicrobial Assays

**Figure 5:**
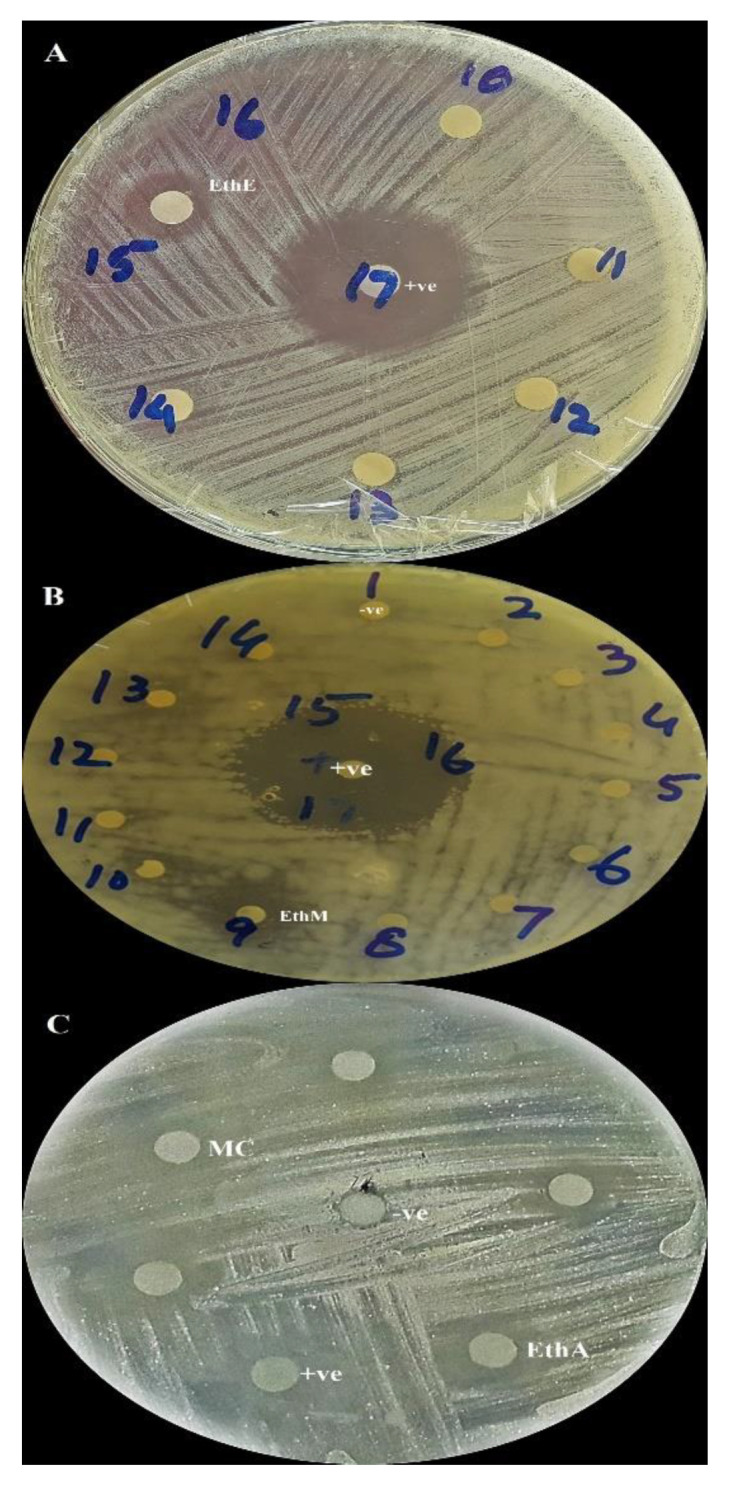
Antibacterial (**A**), antifungal (**B**), and protein kinase inhibition (**C**) representation of selected test samples and controls. EthE = ethyl acetate−ethanol, EthM = ethyl acetate−methanol, EthA = ethyl acetate–acetone, and MC = methanol–chloroform extracts, respectively.

Test extracts were tested against five bacterial species, but they have only shown activity against *P. aeruginosa.* Maximum zones of inhibition (ZOI) were displayed by EthA, Eth, and EthE (Figure 5) with corresponding values of 15 ± 2 mm, 15 ± 1 mm, and 15 ± 1 mm with MIC values of 66.66 µg/mL in all three extracts (Table 4). The DMSO impregnated disc did not form any ZOI.

Results of the antifungal assay are shown in Table 4. Largest ZOI was given by EthM (25 ± 2 mm and MIC of 50 µg/disc) (Figure 5) against *Mucor sp*. The M extract also showed good activity against *A. flavus* (12 ± 1 mm) and *A. fumigatus* (15 ± 2 mm) with a MIC of 100 µg/disc each. When tested against *F. solani*, maximum ZOI were given by MC and EthE with values 16 ± 1 mm and 16 ± 1 mm and MIC values of 100 µg/disc, respectively.

Screening revealed remarkable antileishmanial potential in the Nh extract with % inhibition of 97.5% and 50% mortality (IC_50_) of 120.8 ± 3.7 µg/mL. Thirteen out of the total fourteen extracts showed promising results, which are given in the decreasing order as Nh > EthM > E > EthA > EC > C > DA > EthE > A > Eth > M > MC > DM (Table 4).

#### 3.2.3. Cytotoxicity Assays

Cytotoxicity determination via brine shrimp lethality assay showed the Eth extract to be most potent with a LD_50_ of 29.94 ± 1.6 µg/mL. It was then followed by DA and EthA with a LD_50_ of 48.01 ± 3 µg/mL and 48.75 ± 2.9 µg/mL, respectively (Table 5).

An excellent cytotoxic activity paved the way to also test the plant extracts against the human leukemia (THP-1 ATCC# TIB-202) cell line. A noticeable inhibition in cell line proliferation was discovered in DA and D extracts at 200 µg/mL (Table 5). DA extract exhibited 80.3 ± 2.4% inhibition with IC_50_ of 118 ± 3.4 µg/mL, whereas the D extract showed 64.5 ± 1.4% with an IC_50_ of 140 ± 3.2 µg/mL.

Cytotoxicity in normal isolated human lymphocytes was also checked to assess if the test extracts were selectively toxic to cancer cells or not. None of the extract gave cytotoxicity against normal cells. All extracts provided IC_50_ values > 200 µg/mL (Table 5).

#### 3.2.4. Protein Kinase Inhibition Assay

Inhibitors of protein kinases are represented as distinguished entities that might be facilitated to discover new cancer therapeutic agents. MC (Figure 5) extract exhibited maximum bald ZOI with 19 ± 1 (Table 5). Except for the pure n-hexane and water extracts, all other extracts acted as resourceful prospects to obtain propitious inhibitors of protein kinases.

## 4. Discussion

We observed that the extract yield changed when changing the solvent (Table 1); one possible explanation is the solubility or insolubility of distinct phytochemical constituents in each characteristic solvent [16], which results in different pharmacological responses. First time execution of water–acetone extract regarding its phytochemical analysis was conducted and resulted in alignment with the reported findings where most polar methanol extracts of *C. tuberculata* displayed the maximal result [17]. The existence of various functional groups in the phenolics class (i.e., hydroxyl, methoxy, ketonic, or double bond conjugation) are considered answerable for antioxidant potential [18], in addition to other oxygenated derivatives that also have the same abilities [19]. Ethnomedicine also claims the use of the decoction and infusion of *C. tuberculata* for various ailments [20]. Equilibration of oxidative imbalance by phenols might be due to the presence of flavonoids. The main motive of HPLC analysis is to detect and quantify unreported polyphenols in the study plant. The presence of rutin and gallic acid are perhaps determinants of the known bio-assay results of our study (Table 3; Figure 2). Rutin is known for its compelling health benefits in obesity, diabetes, oxidant imbalance, cancer, and susceptibility to inhibit different bacteria while gallic acid has been proven to be a healing and defensive agent in ameliorating disturbance caused by disturbed redox reactions in cells causing neurodegeneration, cancer, aging, and cardiac problems. [21]. Its indigenous use in treatment of cancer, when taken orally as a dried and ground powder with water or as salad [22], is also strengthened by our findings. Rutin and gallic acid detected in *C. tuberculata* extracts might be considered responsible for antioxidant, antimicrobial, antileishmanial, cytotoxic, and protein kinase inhibitory potential. Polar extracts, due to their significantly higher detection of polyphenols, are recommended for further advance studies related to phytochemistry and biological activity determination, specifically acetone medium extracts alone or in combination with other solvents.

Defense and repair system in response to oxidative damage is innately present in almost every living creature, nevertheless, these defensive mechanisms are insufficient to provide complete protection by oxidative imbalance [23]. Antioxidant actions of an extract cannot be fully determined by a single technique, therefore, an array of different procedures have been used to uncover the antioxidant capacity based on different mechanisms (i.e., free radical scavenging, inhibition of chain initiation, and peroxide decomposition). The decolorization of the DPPH reagent is due to the presence of antioxidants in the test extracts, which can be quantified by computing changes in absorbance by a spectrophotometer [24]. The assay findings conform with previous studies in which the M (most polar solvent used) extract of *C. tuberculata* exhibited the maximum activity, C extract provided comparatively less results than the M extract, and Nh provided the least results [17]. In the phosphomolybdenum based antioxidant assay, the computed results strengthen previous reports that have documented the maximum antioxidant potential by other means in the M extract [25]. The reducing efficacy of plants has been documented to be correlated with the presence of phenolic constituents. The HPLC graphs denoted the presence of phenolics in the test samples. There are different types of compounds called reductones that are responsible for antioxidant potential. Their mechanism of action is either that their peroxide synthesis is hindered or free radical chain breakage by donation of a hydrogen atom [26].

In the antibacterial assay, ethyl acetate extracts, whether alone or in 1:1 proportion with other solvents, proved to be effective only against *P. aeruginosa*. The results might be due to inhibition of protein kinase activity noted in the ongoing investigation, which may act as new antimicrobials [27]. *P. aeruginosa* infections in subcutaneous nodules of immunocompromised patients portend higher risk of mortality and morbidity [28]. Ethnopharmacology claims the use of under discussed plants for skin diseases [29]. Therefore, ethyl acetate combinatorial extracts are suggested to scientifically augment ethnopharmacological data regarding derma. Results are also in accordance with previous studies in which food plants have shown antibacterial activity for Gram-negative bacteria in contrast to medicinal plants that are more potent against Gram-positive bacteria [30].

Fungal infections have drastically increased in immunocompromised patients, which has increased the urgency for newer and safer antifungal agents [31]. Test extracts exhibited well to moderate results when tested against fungal strains. Maximum result was shown against *F. solani*. *Fusarium* skin infection is associated with painful erythematous nodules and papules [32]. Polar extracts of the study plant orally or in the form of cream can be used for fungal infections. Previous studies not providing results against fungal strains is due to the utilization of a unipolarity solvent (i.e., ethanol). Ethanol extracts contain sugars, which promote fungus growth and interferes with the activity of antifungal constituents in extracts [33]. Detailed analysis is required to perceive an exact mode of action and the relationship between fractionation and its effect on biological activity.

Failure of first line therapy and propagation of leishmaniasis worldwide has persuaded researchers to discover new drugs. The findings conform with studies on the closely related specie *Caralluma sinaica*, whose methanolic extract is potent against *L. infantum* [34] and the methanolic extract of *Caralluma quadrangular*, which also showed meaningful activity against *L. infantum* [35]. The assay results also revealed and suggest under discussed extracts as having a possible preventive and supportive role in leishmaniasis. All extracts exhibited meaningful results against *L. tropica*. Oral administration of extracts and/or powder is suggested to be validated by in vivo studies to authenticate the preliminary claim.

It is commonly inferred that brine shrimps or *Artemia salina* larvae and carcinoma cells of mammals behave similarly in many regards, which is why the cytotoxic effects of the undertaken test extracts might become potential candidates for anti-tumor and anti-cancer activities [36]. It is an effective procedure to hypothesize possible biological activities of test extracts against malarial parasites, pests, tumors, and harmful microbes [37]. The pattern of results was also almost observed during the protein kinase inhibition assay. The findings correspond with previous reports in which significant in vitro anticancer results were exhibited by C, Eth (maximum activity), and M, while the Nh and D extracts did not exhibit any activity [11]. Acceptability of *C. tuberculata* as a food despite its high cytotoxic profile is due to the existence of flavonoids and saponins. Dietary flavonoids are known to be involved in reducing RNA and increasing cytotoxicity and mutagenicity. On the other hand, saponins possess strong antimutagenic activity (protection of DNA and protein biosynthesis) and might be considered responsible for the efficacy of herbal drugs. Previous studies have shown *C. tuberculata* as cytotoxic, but not clastogenic in mice [38]. Further research is needed to evaluate the exact mechanism behind the DNA protective effect of this plant specie.

Excellent cytotoxic activity has paved the way to also testing plant extracts against human leukemia (THP-1 ATCC# TIB-202) cell line. Cytotoxicity against THP-1 human leukemia cell line is in accordance with previous studies in which the M (same polarity as acetone) extract exhibited anticancer potential against Caco-2 cells, MCF-7, and MDA-MB-468 cells [11]. These findings can also be related to the undertaken extract testing against brine shrimp lethality and the protein kinase inhibition assay where DA and A extracts have shown prominent results. The findings also conform with the total reducing and total antioxidant capability assays in which the DA extract showed the maximum results, which leads to possible antioxidant cytotoxic activity. Rutin is a flavonoid known for its multispectral pharmacological responses (e.g., hypertension, diabetes, and cancer [39]) and its efficacy has been proven against THP-1 leukemia cells [40]. Therefore, the rutin detected in HPLC graphs of water and methanol extracts may be considered responsible for the noted cytotoxic activity. Moreover, the test extracts did not show cytotoxicity in normal lymphocytes, which ensures targeting the cancer cells only and limiting toxicity toward normal cells. Its indigenous use in the treatment of cancer, when taken orally as dried and ground powder with water or as salad [22], is also strengthened by our findings. Further evaluation needs to be conducted to examine the effects on gene expression/pathway in an animal/human model. However, the sequential extraction method is recommended to remove the non-polar constituents and use only the polar extracts for anticancer use, especially in leukemia.

Protein kinase inhibitory activity might be due to the presence of saponins previously detected in C, Eth, and M extracts [17], which have the potential to downregulate protein kinase C (PKC) [41]. Another possible reason is the presence of gallic acid, as confirmed in HPLC profiling (Figure 2; Table 3), which has anticancer properties against human osteosarcoma cells by interfering with signaling pathways involving protein kinases [42]. Over the years, discovery and development of protein kinase inhibitors have increased from plant sources due to their beneficial aspects [43]. The number of kinases coded in human genome has reached about 500. Test extracts that have the ability to adhere allosterically with either active or inactive site of any of these kinases can be regarded as a potential and crucial source to establish new therapy regimens against cancer [44]. This assay is advantageous in many regards as it quickly identifies a compound as cytotoxic and can also pinpoint potential inhibitors, which hinders signal transduction in infections and cancers [45].

## 5. Conclusions

Dependency of phytochemicals and bioactivities on the polarity of the extraction solvent was observed. Detection and quantification of phenolics, flavonoids, rutin, and gallic acid suggest that *C. tuberculata* is a useful prospect for antioxidants. Significant responses were given by test extracts in in vitro antifungal, antileishmanial, brine shrimp cytotoxicity, THP-1 leukemia cell line cytotoxicity, and protein kinase inhibition assays. Findings revealed pharmacological features of *C. tuberculate*, which authenticates its therapeutic potential in various ailments besides inferring its resourcefulness to isolate phytotherapeutical constituents through bioactivity guided isolation. Our results show that *C. tuberculate* is a worthy lead for advanced studies.

## Figures and Tables

**Table 1:** Gradient volume of mobile phase B (%) added in the system.

Time (min)	%B	Flow Rate (mL)	Max Pressure
0	0	1	350
20	50	1	350
25	100	1	350
30	100	1	350
35	0	1	350
40	0	1	350

**Table 2:** Extract recovery.

S. No.	Solvent Extract Code	% Extract Yield
1	Nh	0.86
2	C	6.76
3	A	10.02
4	EthA	7.12
5	Eth	4.44
6	EC	9.04
7	MC	10.34
8	EthE	10.2
9	EthM	8.70
10	E	12.10
11	DA	16.08
12	M	13.78
13	DM	9.70
14	D	25.16

**Table 3:** RP HPLC analysis of various solvent extracts of *C. tuberculata*.

Extract Name	Polyphenols (µg/mg Extract)	
	Rutin	Gallic Acid
M	0.51	0.35
E	----	----
Eth	----	----
EthM	0.58	----

----: not detected.

**Table 4:** Antibacterial, antifungal activity, and MIC values of test extracts.

Antibacterial Assay			Antifungal Assay							
Extract Names	Diameter of ZOI mm (Mean ± SD) (MIC: µg/mL)		Diameter of ZOI in mm (Mean ± SD) (MIC: µg/disc)							
	*P. aeruginosa*	MIC	*Mucor sp.*	MIC	*F. solani*	MIC	*A. Flavus*	MIC	*A. fumigatus*	MIC
Nh	11 ± 1 ^cd^	--	--	--	--	--	--	--	--	--
C	9 ± 1 ^d^	--	--	--	--	--	--	--	--	--
A	12 ± 1 ^c^	200	--	--	--	--	--	--	--	--
EthA	15 ± 2 ^b^	66.66	--	--	10 ± 1 ^d^	--	--	--	--	--
Eth	15 ± 1 ^b^	66.66	--	--	12 ± 1 ^c^	100	11 ± 1 ^bc^	--	12 ± 1 ^c^	100
EC	9 ± 1 ^d^	--	--	--	8 ± 1 ^e^	--	9 ± 1 ^cd^	--	8 ± 1 ^d^	--
MC	12 ± 1 ^c^	200	--	--	16 ± 1 ^b^	100	8 ± 1 ^d^	--	--	--
EthE	15 ± 1 ^b^	66.66	--	--	16 ± 1 ^b^	100	--	--	--	--
EthM	11 ± 1 ^cd^	--	25 ± 2 ^b^	50	11 ± 1 ^cd^	--	--	--	--	--
E	8 ± 1 ^d^	--	12 ± 2 ^c^	100	--	--	10 ± 1 ^c^	--	14 ± 2 ^b^	100
DA	--	--	--	--	--	--	10 ± 1 ^c^	--	12 ± 1 ^c^	100
M	--	--	--	--	13 ± 1 ^c^	100	12 ± 1 ^b^	100	15 ± 2 ^b^	100
DM	--	--	15 ± 1 ^c^	100	10 ± 1 ^d^	--	12 ± 1 ^b^	100	12 ± 1 ^c^	100
D	--	--	--	--	--	--	--	--	--	--
Cefixime	19 ± 1 ^a^	1.11	--							
Cipro **	19.5 ± 1 ^a^	3.33	--	--	--	--	--	--	--	--
Clotri *			35 ± 2 ^a^							
DMSO	--	--	*--*	--	--	--	--	--	--	--

The sample concentration was 200 µg per disc. Values (mean ± SD) were the average of the triplicate of each plant extract (*n* = 1 × 3). -- = No activity in disc diffusion assay or not applicable (zone < 12 mm) for MIC determination. Cefixime and ** Ciprofloxacin: antibacterial assay positive controls (20 µg/disc); * Clotrimazole: antifungal assay positive control, negative control: DMSO. ^a–e^ represent the significance of results. Samples that display different alphabets were significantly different at *p* < 0.05.

**Table 5:** Leishmania, brine shrimp, THP-1, lymphocyte toxicity studies, and protein kinase inhibition assay.

	Antileishmanial (µg/mL)		Brine Shrimp Cytotoxicity (µg/mL)		THP-1 Cytotoxicity(µg/mL)		Lymphocyte Cytotoxicity (µg/mL)		Protein Kinase Inhibition (µg/disc)	
Samples	% Mortality	LC_50_	% Mortality	LD_50_	% Inhibition	IC_50_	% Inhibition	IC_50_	Diameter (mm) at 100 µg/disc	
	200		200		200		200		Clear Zone	Bald Zone
Nh	97.5 ± 1.1	120.8 ± 3.7 ^b^	0	0	16.4 ± 1.1	>200	6.2 ± 1.1	>200	--	--
C	82.5 ± 1.3	139.1 ± 4.2 ^cd^	100	50.09 ± 2.2 ^c^	21.3 ± 1.7	>200	5.9 ± 1.3	> 200	--	14 ± 0.57 ^c^
A	80.3 ± 2	142 ± 5.7 ^d^	100	98.6 ± 3.4 ^e^	30 ± 2.0	>200	6.6 ± 2.1	>200	--	17 ± 0.57 ^b^
EthA	83.4 ± 2.1	137.6 ± 6.1 ^cd^	100	48.75 ± 2.9 ^c^	19.6 ± 2.1	>200	7.1 ± 1.5	>200	--	14 ± 0.57 ^c^
Eth	80.4 ± 1.8	142 ± 5.9 ^d^	100	29.94 ± 1.6 ^b^	21.7 ± 0.7	>200	7.4 ± 1.1	>200	--	13 ± 1 ^c^
EC	83 ± 1.2	137.6 ± 3.9 ^cd^	100	50 ± 3.01 ^c^	24.2 ± 1.5	>200	5.5 ± 0.9	>200	--	12 ± 1 ^cd^
MC	79 ± 2.2	143.5 ± 6.2 ^de^	100	74.22 ± 3.4 ^d^	26.8 ± 0.8	>200	5.7 ± 1.1	>200	--	19 ± 1 ^b^
EthE	81 ± 1.0	140.5 ± 3.5 ^cd^	100	49.16 ± 2.8 ^c^	22.1 ± 1.5	>200	6.7 ± 1.3	>200	--	10± 1 ^d^
EthM	91 ± 2.2	127.4 ± 6.3 ^bc^	100	49.15 ± 2.7 ^c^	27.5 ± 1.3	>200	6.8 ± 1.6	>200	--	11 ± 0.57 ^cd^
E	85 ± 2.1	134.9 ± 6.0 ^c^	80 ± 5.77	102.24 ± 4.7 ^e^	31 ± 2.8	>200	4.4 ± 0.83	>200	--	10 ± 1 ^d^
DA	82 ± 1.2	139.1 ± 3.5 ^cd^	100	48.01 ± 3.0 ^c^	80.3 ± 2.4 ^b^	118 ± 3.4 ^b^	4.6 ± 1.3	>200	--	8 ± 1 ^de^
M	80 ± 1.8	142 ± 4.3 ^d^	90 ± 5.77	51.34 ± 3.5 ^c^	27.8 ± 2.2	>200	5.8 ± 2.1	>200	--	8 ± 1 ^de^
DM	78 ± 2.3	145.1 ± 6.3 ^e^	90 ± 5.77	50.32 ± 3.3 ^c^	31.7 ± 1.4	>200	4.9 ± 1.2	>200	--	7 ± 0.57 ^e^
D	0	0	30 ± 10	≥200	64.5 ± 1.4 ^c^	140 ± 3.2	4.1 ± 0.66	>200	--	--
Amphoterecin B	100	0.016 ^a^								
Doxorubicin			100	6.24 ± 0.7 ^a^						
5-Florouracil					100	5.3 ± 0.68				
Vincristine					100	8.5 ± 0.92	74.49 ± 3.35	6.6 ± 0.2		
Surfactin										29 ± 1 ^a^
DMSO									-	-
1% DMSO in PBS/sea water	-		-		-		--			

Values are presented as mean ± standard deviation of triplicate analysis. --: no activity. ^a–e^ represent the significance of results. Samples that display different alphabets were significantly different at *p* < 0.05.

## Data Availability

Data is contained within the article.

## Sample Availability

Samples of the plant material are available from the authors.
